# Editorial: Psychiatric disorders of chronic physical diseases

**DOI:** 10.3389/fpsyt.2023.1205336

**Published:** 2023-06-26

**Authors:** Dan Song, Doris Y. P. Leung, Jojo Y. Y. Kwok, Xianliang Liu

**Affiliations:** ^1^College of Nursing, Florida State University, Tallahasse, FL, United States; ^2^School of Nursing, Faculty of Health and Social Sciences, Hong Kong Polytechnic University, Kowloon, Hong Kong SAR, China; ^3^School of Nursing, The University of Hong Kong, Pokfulam, Hong Kong SAR, China; ^4^College of Nursing and Midwifery, Charles Darwin University, Brisbane, QLD, Australia

**Keywords:** psychiatric disorders, physical diseases, co-morbid, editorial/personal viewpoint, multidisciplinary

This Research Topic focuses on co-morbid psychiatric disorders in old adults with Chronic Non-Communicable Diseases (NCDs). The worldwide population is aging rapidly. Between 2015 and 2050, the proportion of the population aged over 60 years worldwide will approximately double from 12 to 22% (1). There is a progressive shift in the burden of NCDs, including cardciovascular diseases, chronic obstructive pulmonary disease, Alzheimer's Disease, etc., (2). These challenges not only affect the patients' physical health but to their mental health as well. Nearly one-third of patients with NCDs manifest with psychiatric disorders such as depressive symptoms or anxiety (3). Co-morbidity of physical and mental disorders will trap the patients in a vicious circle (4). This Research Topic aims to elicit the professional dialogue between the multidisciplinary teams, in order to foster vigorous studies of evidence-based care in diagnosing and managing co-morbid psychiatric symptoms in older adults with NCDs.

In this Research Topic, 49 researchers examined a range of topics impacting mental wellbeing of older people with NCDs. This Research Topic includes a range of methodologies and article types, including national surveys, a bibliometric analysis, qualitative interviews, a systematic review, translation and validation of measurement tool. These papers focus on obtaining a comprehensive understanding of the association between NCDs and psychiatric disorders.

Zhou et al. used bibliometric analysis to reveal the research trends on mental health and multimorbidity in the aging population, with the timeframe set between 2002 and 2022. In their study, over 200 studies were included for analysis. Depressive symptoms and anxiety were identified as the most investigated Research Topics in mental wellbeing in elders with multimorbidity. Research trends lie in risk factors of prognoses and effective interventions for prevention and treatment of psychiatric disorders. They argued that given the bidirectional relationship between mental wellbeing and multimorbidity in elders, further exploration in this field is needed to inspire more beneficial research to promote holistic wellbeing in the aged adults.

Wang et al. conducted a community-based cross-sectional analysis to examine the prevalence and correlates of depression among community-dwelling elders. Their regression model indicated that frailty, living activity dependence, being old, and living alone was independent risk factors for depression incidence among older adults. The results revealed the critical roles of physical frailty and physical function on mental wellbeing. The study reinforced the importance of psychosocial support for older adults who are living alone and with impaired physical health.

Frailty as an emerging concept in gerontology, is a prominent aging-related symptom, and is very common in older adults. Frail older adults had a significant lower quality of life, as well as lower mental wellbeing in older adults. Two studies in this issue examined the correlates of frailty in older adults. Fu et al. found that secondhand smoke is positively associated with increased risk of frailty. Li et al. found that worse cognitive function is also associated with increased risk of frailty. Prevention and reduction of secondhand smoke and management of cognitive decline in older adults may help protect them from developing frailty, and improve their physical and mental wellbeing.

Post-stroke depression is common and associated with poorer recovery and lower quality of life than stroke without depression. Physical exercise rehabilitation is a very effective way to relieve depressive symptoms and improve the disease prognosis. Zhang et al. conducted a qualitative study to explore the influencing factors of home exercise adherence in post-stroke older adults. The study finding indicated that the correlates of home exercise adherence included self-related factors (physical impairment, self-efficacy, and depression), family-related factors (caregiving skills and emotional support), and environment-related factors (exercise prescription, monitoring and feedback, and agency policy). They suggested that healthcare professionals need to assess the patients' physical function and depressive symptoms, build a multi-support system, provide individualized exercise prescription, focus on the monitoring and feedback of exercise rehabilitation, so as to improve the home exercise adherence in elderly patients with stroke.

Research evidence indicates that meeting the psychosocial needs of older adult with cognitive impairment to participate in meaningful activities can reduce their behavioral and psychological symptoms, and improve the quality of life. Because there is a lack of validated scales available in China, Chen et al. translated the Meaningful and Enjoyable Activities Scale (MEAS) into Chinese and tested its reliability and validity in older adults with mild dementia. After translation and cross-cultural debugging, the original version of MEAS has been successfully introduced into China. It provides an effective measurement tool for improving the active engagement of older adults with mild dementia and provides a basis for subsequent research on targeted intervention to improve the psychosocial wellbeing of older adults with mild dementia and delay the progression of cognitive decline.

As expected, mental health in older adults with NCDs has attracted considerable attention in the scientific community. The studies reported in this Research Topic add evidence to the existing literature on the impacts of chronic physical disorders on mental health, thus highlighting the importance of assessment of mental health and psychosocial support for older adults with impaired physical health. Future lines of research should aim to provide more rigorous evidence on effective interventions to manage psychiatric disorders in older adults with NCDs.

## Author contributions

All authors listed have made a substantial, direct, and intellectual contribution to the work and approved it for publication.
